# Nano-Zn Increased Zn Accumulation and Triglyceride Content by Up-Regulating Lipogenesis in Freshwater Teleost, Yellow Catfish *Pelteobagrus fulvidraco*

**DOI:** 10.3390/ijms21051615

**Published:** 2020-02-27

**Authors:** Shi-Cheng Ling, Mei-Qin Zhuo, Dian-Guang Zhang, Heng-Yang Cui, Zhi Luo

**Affiliations:** 1 Key Laboratory of Freshwater Animal Breeding, Ministry of Agriculture, Fishery College, Huazhong Agricultural University, Wuhan 430070, China; 15907174014@webmail.hzau.edu.cn (S.-C.L.); zmq@mail.hzau.edu.cn (M.-Q.Z.); ZDG@webmail.hzau.edu.cn (D.-G.Z.); cuihengyang@webmail.hzau.edu.cn (H.-Y.C.); 2 Laboratory for Marine Fisheries Science and Food Production Processes, Qingdao National Laboratory for Marine Science and Technology, Qingdao 266237, China

**Keywords:** nano-Zn, clathrin pathway, PPARγ, lipid metabolism, vertebrates

## Abstract

The present study was conducted to explore the mechanism of nano-Zn absorption and its influence on lipid metabolism in the intestine of yellow catfish *Pelteobagrus fulvidraco*. Compared to ZnSO_4_, dietary nano-Zn addition increased the triglyceride (TG) content, enzymatic activities of malic enzyme (ME) and fatty acid synthase (FAS), and up-regulated mRNA levels of *6pgd*, *fas*, *acca*, *dgat1*, *pparγ*, and *fatp4*. Using primary intestinal epithelial cells of yellow catfish, compared to the ZnSO_4_ group, nano-Zn incubation increased the contents of TG and free fatty acids (FFA), the activities of glucose-6-phosphate dehydrogenase (G6PD), 6-phosphogluconate dehydrogenase (6GPD), ME, and FAS, up-regulated mRNA levels of lipogenic genes (*6pgd*, *g6pd*, *fas*, *dgat1*, and *pparγ*), genes of lipid transport (*fatp4* and *ifabp*), and Zn transport genes (*znt5, znt7, mt,* and *mtf1*), and increased the protein expression of fatty acid transport protein 4 (FATP4) and peroxisome proliferator activated receptor gamma (PPARγ). Further studies found that nano-Zn absorption was via the clathrin-dependent endocytic mechanism. PPARγ mediated the nano-Zn-induced increase in TG, and nano-Zn increased Zn accumulation and induced TG accumulation by activating the PPARγ pathway and up-regulating lipogenesis.

## 1. Introduction

Zinc (Zn) is an essential nutrient required in animals for many important biological processes, including growth, development, and nutrient metabolism [1]. Dietary Zn deficiency or excess adversely affects biochemical processes and growth in fish. In vertebrate animals, the absorption of Zn mainly occurs in the intestinal tract [2]. Adequate Zn absorption from the intestine is essential for the body, but a high level of Zn absorption will be harmful [2]. Therefore, maintaining an appropriate Zn level is critically important. Zn uptake and the regulation of homeostasis are achieved through many key proteins and genes [3]. These proteins consist of metal-response transcription factor-1 (MTF-1), metallothionein (MT), and transmembrane transporters (ZIP and ZnT families) [4]. MTF-1 is the only Zn-sensing transcription factor in vertebrates and regulates the transcription of many genes involved in Zn metabolism [3]. MT is important for cytosolic Zn storage. ZIP transporters, such as ZIP4, play prominent roles in the Zn uptake and transport of Zn from outside the cell into the cytoplasm [3]. ZnT transporters, such as ZnT1, ZnT5, and ZnT7, mobilize Zn from the cytosol into the extracellular space and the lumens of intracellular compartments [3,5,6]. The regulation of dietary Zn absorption in the intestine is believed to be important in Zn homeostasis and has been studied in rodent models, but not in fish. The molecular mechanism for Zn uptake and homeostatic regulation remains unknown in fish.

As Zn is an essential microelement in fish, many studies have been performed to determine the effects of dietary Zn levels on growth performance, body composition, and nutrient metabolism [7,8]. In our laboratory, studies have suggested that dietary Zn addition significantly influence lipid deposition and metabolism by regulating enzymatic activities and genes expression, including lipogenic enzymes and genes: Glucose-6-phosphate dehydrogenase (G6PD), 6-phosphogluconate dehydrogenase (6GPD), acetyl-CoA carboxylase (ACC), fatty acid synthase (FAS), and transcription factors peroxisome proliferator-activated receptor gamma (PPARγ) and sterol regulatory element binding protein-1 (SREBP-1), which, in turn, affects health and fillet quality [9]. In these studies, inorganic Zn has been used as an additive in the diets. However, low utilization efficiency is the biggest issue for its application because low Zn utilization in fish leads to excess Zn excretion in the aquatic environment and causes environmental pollution. Meanwhile, studies have suggested that its effects of dietary Zn levels depend on the chemical forms of Zn because its chemical form affects Zn absorption and utilization [10,11]. Zn nanoparticles (nano-Zn), as a substitute to the conventional Zn sources, can be a good alternative in aquatic feed. It has been reported that nanoparticles showed new characteristics of transport and uptake and exhibited a higher absorption efficiency [10,11]. Thus, nano-Zn may be used in feed to provide better results than inorganic Zn sources and indirectly prevent environmental contamination. Zn nanoparticles have been reported to enhance growth performance and improve feed utilization and many other beneficial effects in fish [12,13]. However, the cellular mechanisms of these particles are not well understood. In mammals, it has been reported that Zn nanoparticles are internalized in cells via the endocytic pathway. Endocytosis is a conserved process in eukaryotes by which extracellular components are taken up into cells by invagination of the plasma membrane to form vesicles that enclose these materials [14]. There are four main endocytic pathways for internalizing nanoparticles, such as macropinocytosis, clathrin-dependent, caveolae-dependent, and clathrin-caveolae-independent endocytosis [14,15]. Understanding which of these pathways are involved in nano-Zn uptake is critical to clarifying the regulatory mechanism of nano-Zn in fish.

As a continuation of our series of studies involved in Zn nutrition and its relationship with lipid metabolism in fish, the present study was conducted to compare the effects and mechanism of dietary ZnSO_4_ and nano-Zn addition influencing Zn absorption and lipid metabolism in the intestinal tract of yellow catfish *Pelteobagrus fulvidraco*, an omnivorous freshwater fish widely distributed in China and other Asian countries. Considering that two Zn sources (inorganic and nano-Zn) exist in aquatic environments and aquatic feedstuffs, our study provided important references and mechanistic insights for the evaluation and assessment of Zn nutrition and hazards in vertebrates.

## 2. Results

### 2.1. Growth Performance, Feed Utilization, and Morphological Parameters

Survival, feed intake (FI), and intestinal somatic index (ISI) showed no significant differences between the two groups (Supplementary Table S2). Weight gain (WG), specific growth rate (SGR), viscerosomatic index (VSI), and condition factor (CF) were higher in thenano-Zn groups than in the ZnSO_4_ group. By contrast, feed conversion rate (FCR) was higher in the ZnSO_4_ group than in the nano-Zn group (Supplementary Table S2).

### 2.2. Zn Accumulation, Zn Absorption, and Lipid Metabolism in the Intestine

**Figure 1:**
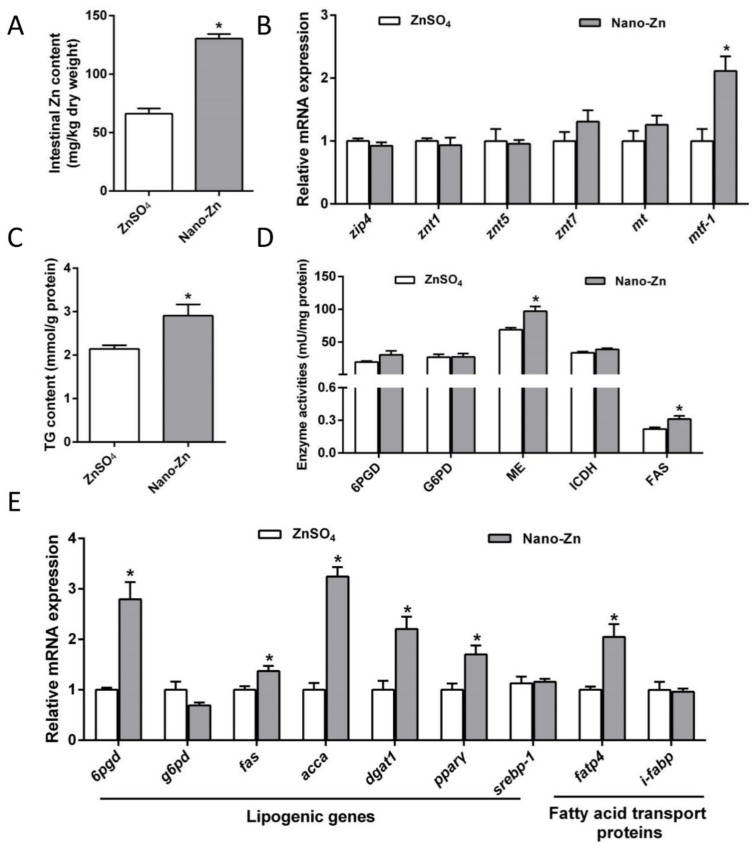
Effects of two Zn sources on Zn transport proteins and lipid metabolism of the intestine of yellow catfish. (**A**) Zn content. (**B**) The mRNA levels of Zn transport protein. (**C**) Triglyceride (TG) content. (**D**) Enzyme activities. (**E**) The mRNA levels of the lipogenic genes. Values indicate means ± SEMs, *n* = 3. The mRNA levels of genes from the ZnSO_4_ group were considered to be 1. Asterisks (∗) indicate significant differences between ZnSO_4_ and the nano-Zn group (*p* < 0.05).

Intestinal Zn content and *mtf-1* mRNA expression were higher in the nano-Zn group than in the ZnSO_4_ group (Figure 1A,B). mRNA levels of *zip4*, *znt1, znt5, znt7*, and *mt* showed no significant differences between the two groups. Compared to ZnSO_4_, nano-Zn increased the triglyceride (TG) content, enzymatic activities of malic enzyme (ME) and FAS, and up-regulated mRNA levels of *6pgd*, *fas*, *acca*, *dgat1*, *pparγ*, and *fatp4* (Figure 1C–E). mRNA levels of *g6pd*, *srebp-1,* and *i-fabp* showed no significant differences between the other two groups.

### 2.3. Intestinal Epithelial Cells Absorb Nano-Zn via Clathrin Pathway

**Figure 2:**
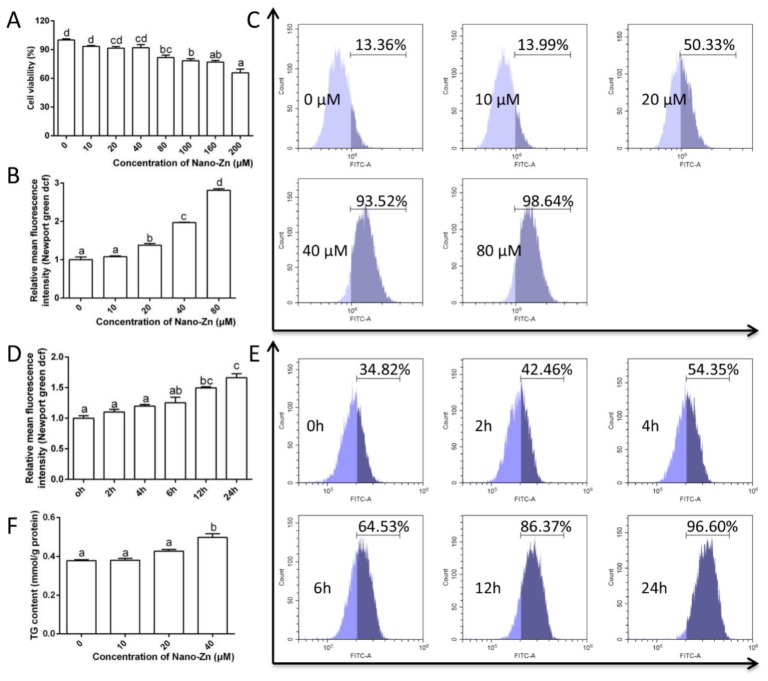
Nano-Zn enhanced the content of free Zn^2+^ and TG accumulation in the intestinal epithelial cells of yellow catfish. (**A**) Cell viability after 24 h of nano-Zn incubation. (**B**) The free Zn^2+^ quantified by calculating FL1 (green) mean fluorescence intensity (Newport Green DCF) after the primary intestinal epithelial cells were incubated with nano-Zn for 24 h. (**C**) The presence of Newport Green DCF-stained Zn^2+^ demonstrated by flow cytometry analysis of green (FL1) fluorescence intensity after the primary intestinal epithelial cells were incubated with nano-Zn for 24 h. (**D**) The free Zn^2+^ was quantified by calculating the FL1 (green) mean fluorescence intensity (Newport Green DCF) after the primary intestinal epithelial cells were incubated with 40 µM nano-Zn. (**E**) The presence of Newport Green DCF-stained Zn^2+^ was demonstrated by flow cytometry analysis of green (FL1) fluorescence intensity after the primary intestinal epithelial cells were treated with 40 µM nano-Zn. (**F**) TG content after 24 h of nano-Zn incubation. Values indicate means ± SEMs, *n* = 3–6. Different letters indicate significant differences among groups at *p* < 0.05.

To explore the mechanism of nano-Zn absorption by intestinal epithelial cells, the primary intestinal epithelial cells from yellow catfish were isolated and several in vitro experiments were conducted. The 3-(4,5-dimethylthiazol-2-yl)-2,5-diphenyltetrazolium bromide (MTT) assay showed that nano-Zn concentrations of lower than 40 µM had no adverse influence on cell viability (Figure 2A). Green fluorescence intensity by a Zn^2+^ fluorescent probe increased with nano-Zn incubation in a concentration- and time-dependent manner (Figure 2B–E). TG content increased with increasing nano-Zn concentration (Figure 2F).

Compared to the ZnSO_4_ group, nano-Zn incubation increased the protein level of MTF1 (Supplementary Figure S1A) and mRNA levels of Zn transport genes (*znt5, znt7, mt*, and *mtf1*), but not the mRNA levels of *zip4* and *znt1* (Supplementary Figure S1B).

**Figure 3:**
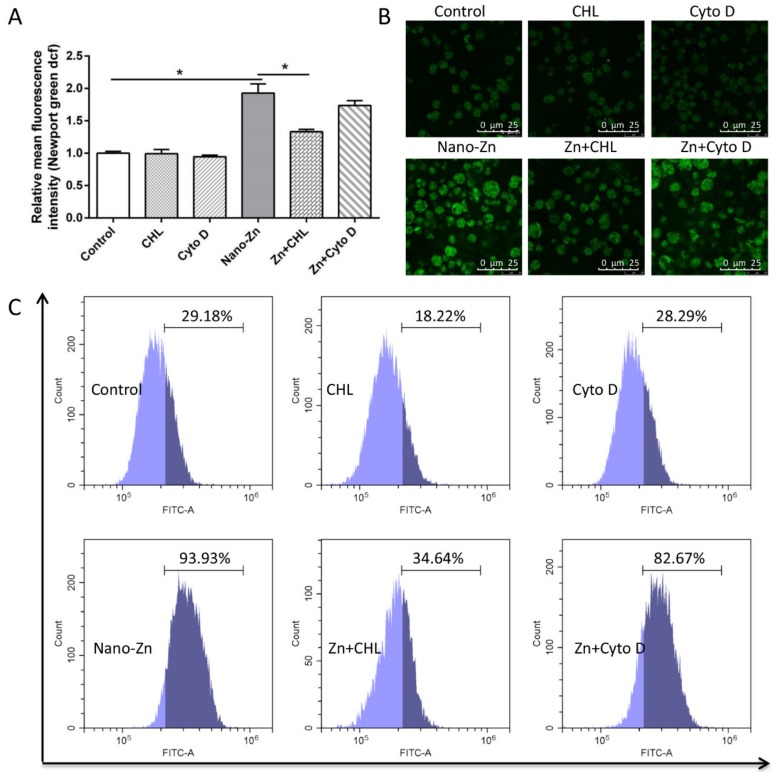
Effect of nano-Zn on clathrin pathway in intestinal epithelial cells. (**A**) Free Zn^2+^ was quantified by calculating the FL1 (green) mean fluorescence intensity of intestinal epithelial cells incubated for 12 h in 40 µM nano-Zn with 2 h of 10 µg/mL CHL or 10 µM cytochalasin D pretreatment. (**B**) Representative confocal microscopy stained with Zn^2+^ fluorescent probe (Newport Green DCF). The primary intestinal epithelial cells from *P. fulvidraco* were incubated for 12 h in control or 40 μM nano-Zn containing medium with or without 2 h of 10 µg/mL CHL and 10 µM cytochalasin D pretreatment. (**C**) The presence of DCF-stained Zn^2+^ was demonstrated by flow cytometric analysis of green (FL1) fluorescence intensity. The primary intestinal epithelial cells from *P. fulvidraco* were incubated for 12 h in control or 40 µM nano-Zn containing medium with or without 2 h of 10 µg/mL CHL and 10 µM cytochalasin D pretreatment. Values indicate means ± SEMs, *n* = 3–6. Asterisks (∗) indicate significant differences between the two groups (*p* < 0.05, *n* = 3). CHL, chlorpromazine; Cyto D, cytochalasin D.

To investigate the role of specific endocytic pathways in the internalization of nano-Zn, primary intestinal epithelial cells were treated with a range of inhibitors: NaN_3_, chlorpromazine (CHL), nystatin, and cytochalasin D (Cyto D). Here, NaN_3_ pretreatment significantly alleviated the Zn uptake of the nano-Zn source, but nystatin showed no significant effects on Zn absorption of the nano-Zn source (Supplementary Figure S2A–C). CHL pretreatment, but not cytochalasin D, significantly alleviated the Zn-induced increase in nano-Zn absorption (Figure 3A–C), indicating that the absorption of the nano-Zn source was via the clathrin pathway.

### 2.4. Higher TG Accumulation in Nano-Zn Group Than in Znso_4_ Group was Attributable to the Nano-Zn-Induced Activation of PPARγ

**Figure 4:**
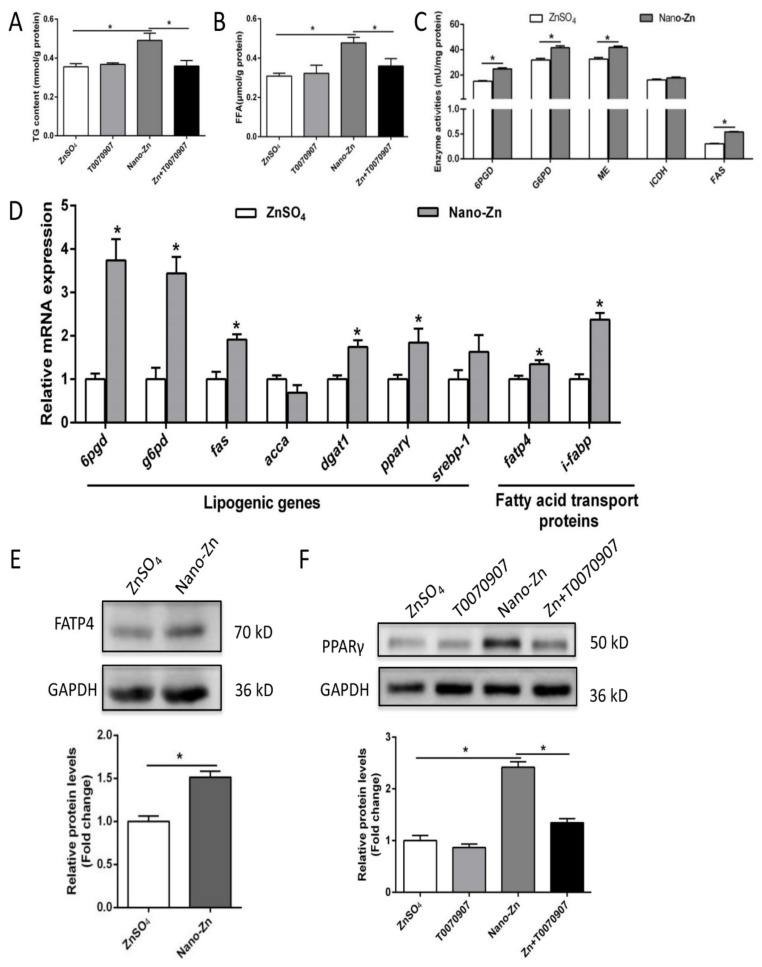
Nano-Zn induced TG accumulation by peroxisome proliferator-activated receptor gamma (PPARγ) pathway in intestinal epithelial cells. (**A**) TG content after 24 h treatments. (**B**) Free fatty acid content after 24 h treatments. (**C**) Enzyme activities. (**D**) The mRNA levels of lipogenic enzymes after ZnSO_4_ or nano-Zn incubation for 24 h. (**E**) Protein levels of fatty acid transport protein 4 (FATP4) after 24 h of 40 µM nano-Zn incubation. (F) Protein levels of PPARγ of intestinal epithelial cells incubated for 24 h in control or 40 µM nano-Zn containing medium with or without 2 µM T0070907 pretreatment for 2 h. Values indicate means ± SEMs, *n* = 3–6. Asterisks (∗) indicate significant differences between two groups (*p* < 0.05, *n* = 3).

Compared to the ZnSO_4_ group, nano-Zn incubation increased the contents of TG and free fatty acids (FFA), the activities of 6PGD, G6PD, ME, and FAS, up-regulated the mRNA levels of *6pgd*, *g6pd*, *fas*, *pparγ*, *fatp4*, *ifabp*, and *dgat1*, and increased the protein expression of fatty acid transport protein (FATP4) and PPARγ (Figure 4A–F). T0070907, a specific inhibitor of PPARγ, alleviated the nano-Zn-induced increase in PPARγ protein expression, and the contents of TG and free fatty acids (FFA), indicating that PPARγ mediated the nano-Zn-induced increase in TG.

**Figure 5:**
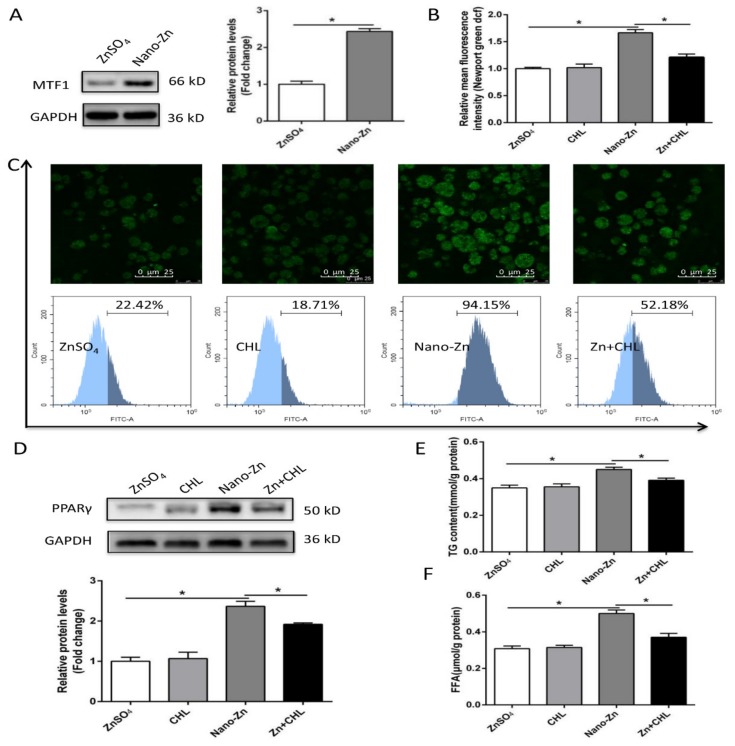
Nano-Zn-induced up-regulation of PPARγ protein levels and TG content is related to nano-Zn absorption. (**A**) Protein levels of MTF1 after 40 µM nano-Zn incubation for 24 h. (**B**) Free Zn^2+^ was quantified by calculating the FL1 (green) mean fluorescence intensity of intestinal epithelial cells incubated for 24 h in 40 µM nano-Zn with 10 µg/mL CHL pretreatment for 2 h. (**C**) Representative confocal microscopy stained with Zn^2+^ fluorescent probe (Newport Green DCF), and the presence of DCF-stained Zn^2+^ was demonstrated by flow cytometric analysis of green (FL1) fluorescence intensity. The primary intestinal epithelial cells from *P. fulvidraco* were incubated for 24 h in ZnSO_4_ or 40 µM nano-Zn containing medium with or without 10 µg/mL CHL pretreatment for 2 h. (**D**) Protein levels of PPARγ of intestinal epithelial cells incubated for 24 h in control or 40 μM nano-Zn containing medium with or without 10 µg/mL CHL pretreatment for 2 h. (**E**) TG content after 24 h treatments. (**F**) Free fatty acid content after 24 h treatments. Values indicate means ± SEMs, *n* = 3–6. Asterisks (∗) indicate significant differences between two groups (*p* < 0.05, *n* = 3). CHL, chlorpromazine.

Next, we use CHL to inhibit the uptake of nano-Zn via the clathrin pathway to determine the effect of nano-zinc on lipid metabolism. Compared to the ZnSO_4_ group, nano-Zn incubation up-regulated the protein expression of MTF-1 and intracellular free Zn^2+^ content (Figure 5A–C). CHL pretreatment alleviated the nano-Zn-induced increase in Zn^2+^ content and alleviated the nano-Zn-induced increase in the PPARγ protein levels and TG and FFA contents (Figure 5C–F).

**Figure 6:**
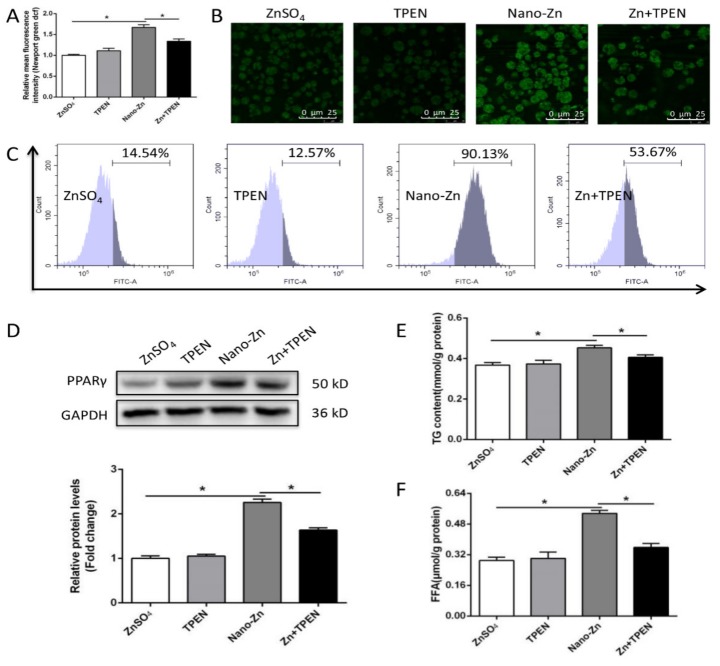
Nano-Zn-induced up-regulation of PPARγ protein levels and TG content is dependent on the release of free Zn^2+^. (**A**) Free Zn^2+^ was quantified by calculating the FL1 (green) mean fluorescence intensity of intestinal epithelial cells incubated for 24 h in 40 µM nano-Zn after 5 µM TPEN pretreatment. (**B**) Representative confocal microscopy stained with Zn^2+^ fluorescent probe (Newport Green DCF). The primary intestinal epithelial cells from *P. fulvidraco* were incubated for 24 h in ZnSO_4_ or 40 µM nano-Zn containing medium with or without 5 µM TPEN pretreatment for 2 h. (**C**) The presence of DCF-stained Zn^2+^ was demonstrated by flow cytometric analysis of green (FL1) fluorescence intensity. The primary intestinal epithelial cells from *P. fulvidraco* were incubated for 24 h in ZnSO_4_ or 40 µM nano-Zn containing medium with or without 5 µM TPEN pretreatment for 2 h. (**D**) PPARγ protein levels of intestinal epithelial cells incubated for 24 h in control or 40 µM nano-Zn containing medium with or without 5 µM TPEN pretreatment for 2 h. (**E**) TG content after 24 h treatment. (**F**) Free fatty acid content after 24 h treatments. Values indicate means ± SEMs, *n* = 3–6. Asterisks (∗) indicate significant differences between two groups (*p* < 0.05, *n* = 3).

To further confirm that nano-Zn-induced changes in TG and FFA were attributable to the change in intracellular free Zn^2+^ levels, we used N,N,N′,N′-tetrakis (2-pyridylmethyl) ethylenediamine (TPEN) to inhibit Zn uptake. TPEN pretreatment alleviated the nano-Zn-induced increase in intracellular free Zn^2+^ (Figure 6A–C), PPARγ protein expression, and the contents of TG and FFA (Figure 6D–F). These observations further indicated that the higher TG accumulation in the nano-Zn group than in the ZnSO_4_ group was attributable to the nano-Zn-induced activation of PPARγ.

## 3. Discussion

In the present study, we found that, compared to the ZnSO_4_ group, dietary nano-Zn promoted growth, increased the Zn and TG content, and up-regulated lipogenesis in the intestine. Then, our in vitro study further found that the absorption of nano-Zn into intestinal epithelial cells via the chathrin-dependent pathway is an energy-consuming process, and Zn^2+^ increased the NEFA and TG contents via the PPARγ pathway. 

In the present study, WG and SGR were higher and FCR was lower in the nano-Zn groups than in the ZnSO_4_ group, indicating that the appropriate concentrations of nano-Zn are better than those of ZnSO_4_ for improving the efficiency of feed utilization and growth performance. Similarly, several studies indicated that Zn nanoparticles improved the production performance than inorganic Zn [16,17].

Sufficient Zn uptake was important for the normal metabolism of fish. The present study indicated that intestinal Zn content and *mtf-1* mRNA expression were higher in the nano-Zn group than in the ZnSO_4_ group. The different Zn contents between the two treatments were probably related to the different absorption process and metabolic pathways. Studies suggested that nano-Zn was more bio-available than inorganic Zn, and accordingly resulted in a higher body Zn concentration [18]. The in vitro study found that the protein level of MTF1 and the mRNA levels of Zn transport genes (*znt5, znt7, mt,* and *mtf1*) were higher in the nano-Zn group than in the ZnSO_4_ group. These data indicated that these key genes and proteins mediated the Zn uptake, transport, and metabolism, in agreement with other studies [6,19]. Studies suggested that nano-Zn can be dissolved in the digestive tract and accordingly release Zn^2+^ [20]. Using artificial digestive liquids (i.e., gastric juice, duodenal juice, and bile), Wang et al. [20] observed initial rapid dissolution (ca. 50%) of nano-ZnOs in both types (acid and alkaline digestion) of digestion liquids within 10 min. Continuous dissolution of nano-ZnOs proceeded in the acid digestion liquid and most (ca. 85%) of the added nano-ZnOs dissolved at 120 min. However, no significant dissolution of nano-ZnOs was observed in alkaline digestion after 10 min [20]. The chemical softness hierarchy may be used in selecting from bi-local, local, and global reactive structures [21], while its inverse chemical hardness hierarchy provides the stability measure [22]. Moreover, Wang et al. [20] speculated that the continuous dissolution of nano-ZnOs in the intestinal lumen would offer highly bioavailable Zn^2+^. Cytoplasmic Zn^2+^ is gauged by the transcription factor MTF-1, which induces transcription of Zn^2+^ chelators such as MTs [4,23], as observed in the present study. Shen et al. [24] pointed out that the MT1 mRNA expression was up-regulated at high Zn concentrations to enhance Zn efflux and, thus, help maintain Zn homeostasis. ZnT5 and ZnT7 localize at the apical membrane of enterocytes, and contribute to the homeostatic maintenance of the secretory pathway functions by supplying Zn into the lumen [3,6,25].

On the other hand, understanding the endocytosis mechanism of nanoparticles is important to help elucidate the mechanism of nano-Zn absorption [26]. The present study indicated that NaN_3_ pretreatment significantly alleviated the uptake of nano-Zn, but nystatin showed no significant effects on the absorption of nano-Zn. NaN_3_ inhibits energy-dependent endocytosis pathways and effectively inhibits the internalization of nanoparticles [26]. This is indicative of the active uptake of the nanoparticles via an energy-dependent process. Similarly, CHL pretreatment, but not cytochalasin D, significantly alleviated the Zn-induced increase in nano-Zn absorption. CHL can prevent clathrin-mediated endocytosis by disrupting the assembly of the clathrin adaptor protein at the cell surface [27]. These observations clearly suggest that clathrin-mediated endocytosis is the principal mechanism for the cellular internalization of nano-Zn in the intestinal epithelial cells, in agreement with other studies [10,14].

Although many studies have found that dietary Zn addition influenced lipid deposition and metabolism [28,29], inorganic Zn has been used in these studies. Different chemical forms of Zn and other minerals will influence the effects. To our best knowledge, this is the first study to explore the effects and mechanism of dietary nano-Zn addition influencing lipid deposition and metabolism in fish. Compared to the ZnSO_4_ group, nano-Zn increased the TG content, enzymatic activities of ME and FAS, and up-regulated mRNA levels of *6pgd*, *fas*, *acca*, *dgat1*, *ppar*γ, and *fatp4*. Our in vitro studies also indicated that, compared to the ZnSO_4_ group, nano-Zn incubation increased the contents of TG and FFA, activities of 6PGD, G6PD, ME and FAS, up-regulated mRNA levels of *6pgd*, *g6pd*, *fas*, *ppar*γ, *fatp4*, *ifabp* and *dgat1*, and increased the protein expression of FATP4 and PPARγ. 6PGD, G6PD, ME, FAS, ACCa, DGAT1, and PPARγ are key enzymes involved in the biosynthesis of fatty acid and TG [9,29]. Thus, our study indicated that the higher intestine TG content in the nano-Zn group was due to the up-regulated lipogenesis. Similarly, our other studies indicated that inorganic Zn induced lipolytic responses [29,30]. Moreover, the present study found that T0070907, a specific inhibitor of PPARγ, alleviated the nano-Zn-induced increase in PPARγ protein expression and the contents of TG and FFA, indicating that PPARγ mediated the nano-Zn-induced increase in TG. PPARγ is critical for the regulation of lipogenesis and promotes lipid storage [31]. Many studies pointed out that T0070907 reduced PPARγ expression at the protein and transcriptional levels, which is in agreement with many other studies [32,33,34]. PPARγ is a key transcriptional factor that regulates lipogenesis, and its reduction in expression levels will, in turn, reduce TG accumulation, as shown in another study [35]. Similarly, Zheng et al. [36] reported a positive correlation between mRNA expression of PPARγ and genes encoding lipogenic enzymes (G6PD, 6PGD, and FAS) in yellow catfish. Here, CHL and TPEN pretreatments, which inhibit nano-Zn uptake, alleviated the nano-Zn-induced increase in the PPARγ protein levels and TG and FFA contents, further indicating that nano-Zn-induced changes in TG and FFA were influenced by Zn. Meanwhile, higher TG accumulation in the nano-Zn group than in the ZnSO_4_ group was attributable to the nano-Zn-induced activation of PPARγ in intestinal epithelial cells.

Here, we characterized the pathway of absorption of nano-Zn into the intestine and elucidated its mechanism of nano-Zn influencing intestinal lipid metabolism. Nano-Zn was absorbed into the intestine via the endocytosis-mediated clathrin pathway. Compared to ZnSO_4_, nano-Zn increased the TG content and up-regulated lipogenesis by activating the PPARγ pathway, which underlies the mechanism of action of nano-Zn for lipid metabolism. Considering that two Zn sources (inorganic and nano-Zn) widely exist in aquatic environments and aquatic feedstuffs, our study provided important references and mechanistic insights for the evaluation and assessment of Zn nutrition and hazards in vertebrates.

## 4. Materials and Methods

Two experiments were performed. The experiment performed on animals and cells followed the ethical guidelines of Huazhong Agricultural University (HZAU) for the care and use of laboratory animals and was approved by the Ethical Committee of HZAU (identification code: Fish-2016-0404, Date: 4 April 2016).

### 4.1. Expt. 1: In Vivo Study

The experimental protocols for yellow catfish culture and management were similar to those described in our recent study [29]. Briefly, two experimental diets were formulated to use ZnSO_4_ (≥99.0% in purity, Sinopharm Chemical Reagent Co. Ltd, Shanghai, China) and Zn nanoparticles (nano-Zn) (average size: 40–60 nm, ≥99% in purity; Sigma) as Zn sources (Supplementary Table S1). Final Zn contents in the experimental diets were determined to be 23.46 and 23.01 mg/kg for the ZnSO_4_•7H_2_O and nano-Zn groups, respectively. At the initiation of the feeding study, 30 uniform-sized fish (mean initial weight: 4.08 ± 0.09 g) were randomly stocked in each fiberglass tank. Each diet was assigned to three tanks in a completely randomized design, with 6 tanks for the experiment. The following values of the parameters of water quality were used: Water temperature from 27.6 °C to 29.9 °C; pH 8.3 ± 0.05; dissolved oxygen 5.79 ± 0.08 mg/L; NH_4_-N 0.11 ± 0.01 mg/L. The experiment continued for 10 weeks.

At the end of the 10 week period, 24 h after the last feeding, all fish were euthanized (MS-222 at 100 mg/L), counted, and weighed to determine survival, WG, and SGR. Then, fish were dissected and the contents of the intestine were gently scraped off. The whole intestine tract was used for the following analysis. A total of four fish per tank were randomly collected and dissected on ice to obtain the intestine samples for TG analysis. For enzyme activity and mRNA expression assays, the intestine samples of twelve fish from each tank (six fish for enzymatic activities and six fish for mRNA expression) were removed immediately using sterile forceps, frozen in liquid N_2_, and stored at −80 °C (not longer than 2 weeks) for further processing. Remaining samples were stored at −80 °C for determining the Zn content.

### 4.2. Expt. 2: In Vitro Study

Intestinal epithelial cells were isolated from yellow catfish based on the methods of our previous studies [4,37]. Nano-Zn was dissolved in ultra-pure water at 10 mM stock concentrations and then sterilized. The nano-Zn solution was treated with an ultrasonic cleaner for 20 min before each use. The primary intestinal epithelial cells from *P. fulvidraco* were incubated for 12 h in 40 µM ZnSO_4_ or 40 µM nano-Zn containing medium with or without 2 h of pretreatment inhibitors. The inhibitors included sodium azide (NaN_3_, 10 mM) for inhibiting energy-dependent internalization pathways, chlorpromazine (CHL, 10 µg/mL), nystatin (5 µg/mL), and cytochalasin-D (5 µg/mL) for inhibiting clathrin-dependent, caveolae-dependent, and phagocytosis-dependent pathways, respectively [14,38]. The concentrations of inhibitors were selected according to our pilot trials and to other in vitro studies [38,39]. They did not adversely influence cell viability. The protein level of MTF1 and the fluorescence intensity of Zn determined by flow cytometry were determined. Each treatment was performed in triplicate.

To determine the mechanism of nano-Zn influencing lipogenesis, chlorpromazine (10 µg/mL), TPEN (Zn^2+^ chelator, Sigma, MO, USA), and T0070907 (PPARγ inhibitor, Selleck, TX, USA) were used. The concentrations of inhibitors were selected according to our previous studies [4,37]. The primary intestinal epithelial cells from *P. fulvidraco* were incubated for 24 h in control or 40 µM nano-Zn containing medium with or without 2 h of pretreatment inhibitors. The protein levels of MTF1, PPARγ, FATP4, and TGn, FFA content, lipid metabolism enzyme activities, gene expressions, and fluorescence intensity of Zn by flow cytometry were determined. Each treatment was also performed in triplicate.

### 4.3. Cell Viability, TG Content, and Enzymatic Activity Assays

The MTT assay was performed to test the cell viability according to our recent protocols [37]. TG content was analyzed by using the glycerol 3-phosphate oxidase p-aminophenol method. The enzyme activities of 6PGD, G6PD, ME, ICDH, and FAS followed the methods described by Wei et al. [29]. One unit of enzyme activity (U), defined as the amount of enzyme that converted 1 µmol of substrate to product per min at 30 °C, was expressed as units per milligram (mg) of soluble proteins. Soluble protein content was analyzed based on the protocols by Bradford [40]. These analyses were undertaken in triplicates.

### 4.4. Real-Time Quantitative PCR (qPCR)

The analysis of gene transcription levels was based on our recent publication [36,37]. The primers are given in Supplemental Table S2. A set of ten housekeeping genes (*18s rrna, β-actin, hprt, b2ma, ubce tuba, gapdh, rpl7, tbp,* and *elfa)* were selected in order to test their transcription stability. The relative expression of genes was calculated using the 2^−ΔΔ*C*t^ method when normalizing to the geometric mean of the best combination of two genes, as analyzed by geNorm [41].

### 4.5. Immunoblotting Analysis

The immunoblotting analysis of MTF1, PPARγ, and FATP4 protein expression followed the methods described by our recent publication [29]. Cell lysates were prepared with Radio-Immunoprecipitation Assay (RIPA) buffer (Thermo Fisher Scientific, Waltham, MA, US). Twenty-five micrograms of protein was separated on 12% sodium dodecyl sulfate (SDS)-polyacrylamide gels. After (sodium dodecyl sulfate polyacrylamide gel electrophoresis (SDS-PAGE), the proteins were transferred to a PVDF membrane, and then blocked with 8% (*w/v*) dry milk for 2 h. The membrane was incubated with antibodies against PPARγ (16643-1-AP, Proteintech, Rosemont, IL, US), MTF1 (25383-1-AP, Proteintech), and FATP4 (11013-1-AP, Proteintech) overnight at 4 °C. Then, they were processed with goat anti-rabbit IRDye 800CW secondary antibody (926-32211; Li-Cor Biosciences, Lincoln, NE, USA). The protein bands were visualized with an Odyssey Infrared Fluorescent Western Blots Imaging System from Li-Cor Bioscience and quantified by Image-Pro Plus 6.0 (Media Cybernetics, Rockville, MD, USA).

### 4.6. Zn^2+^ Measurement

The tissue Zn content was determined by ICP-AES based on the methods by Wu et al. [37]. Newport Green DCF (Ex/Em = 505/535 nm) was used to measure intracellular Zn^2+^ concentrations, based on the methods described in Wei et al. [29].

### 4.7. Statistical Analysis

The results were performed as mean ± SEM. Data were evaluated using one-way ANOVA, and Duncan’s multiple range tests were used to compare the significant differences among more than three treatments. For the comparison between two groups, Student’s *t*-tests were employed. The analysis was carried out using SPSS 19.0, and a *P* value of less than 0.05 was considered significant.

## Supplementary Materials

Supplementary materials can be found at https://www.mdpi.com/1422-0067/21/5/1615/s1.

## Abbreviations

6PGD	6-phosphogluconate dehydrogenase
ACC	acetyl-CoA carboxylase
ANOVA	one-way analysis of variance
CF	condition factor
DGAT	diacylglycerol acyltransferase
FAS	fatty acid synthase
FATP4	fatty acid transport protein 4
FCR	feed conversion rate
FFA	free fatty acid
FI	feed intake
G6PD	glucose 6-phosphate dehydrogenase
ICDH	isocitrate dehydrogenase
I-FABP	intestine fatty acid binding protein
ISI	intestinal somatic index
ME	malic enzyme
MS-222	tricaine methanesulfonate
MT	metallothionein
MTF-1	metal response element-binding transcription factor-1
NaN_3_	sodium azide
PPAR	peroxisome proliferator activated receptor
SEM	standard error of mean
SGR	specific growth rate
SREBP	sterol regulatory element-binding protein
TG	triglyceride
TPEN	N,N,N′,N′-tetrakis (2-pyridylmethyl) ethylenediamine
VSI	viscerosomatic index
WG	weight gain
ZIP	ZRT, IRT-like protein
ZnT	zinc transporter
Zn	zinc
ZnSO4	zinc sulfate
Nano-Zn	zinc nanoparticles
