# Supplementary material for: Nano-Zn Increased Zn Accumulation and Triglyceride Content by Up-Regulating Lipogenesis in Freshwater Teleost, Yellow Catfish Pelteobagrus fulvidraco
Source: Int J Mol Sci. 2020 Feb 27;21(5):1615. doi: 10.3390/ijms21051615 (PMC7084257; doi:10.3390/ijms21051615)
Supplement: Supplementary file 1 [file ijms-21-01615-s001.pdf]

Supplemental Table 1. Feed formulation and proximate analysis of experimental diets.

	$ZnSO_4$	Zn-Nano
Casein	350	350
White fish meal	100	100
Gelatin	20	20
Fish oil	20	20
Soybean oil	40	40
Starch	200	200
Ascorbyl-2-polyphosphate	10	10
NaCl	10	10
$CaH_2PO_4 \cdot 2H_2O$	10	10
Vitamin mix	5	5
Mineral mix (Zn free)	5	5
Zn source	0.035	0.010
Betaine	10	10
Cellulose	219.965	219.99
Moisture	4.05	3.81
Crude ash	3.9	3.77
Crude protein	38.82	38.17
Crude lipid	7.13	7.53
Zn (mg kg^{-1})	23.46	23.01

Supplemental Table 2. Primers used for real-time quantitative PCR analysis

Genes	Forward primer (5’-3’)	Reverse primer (5’-3’)	Accession no.
6pgd	GCTCTGATGTGGCGAGGTGG	CGTAGAAGGACAGTGCAGTGG	JX992745
g6pd	CAGGAATGAACGCTGGGATG	TCTGCTACGGTAGGTCAGGTCC	JX992744
fas	AACTAAAGGCTGCTGGTTGCTA	CACCTTCCCGTCACAAACCTC	JN579124
acca	GGGGTTTTCACGCTGCTTC	GGTTCTGATTGGGTCGTCCTG	JX992746
srebp-1	CTGGGTCATCGCTTCTTTGTG	TCCTTCGTTGGAGCTTTTGTCT	JX992742
pparγ	ACGCCCCGTTCGTTATCC	TGAGCAGAGTCACCTGGTCATTG	JX992741
dgat 1	GCACCATCCACTGCTGTATCA	CGCTCCAACTCTTGTCCGTC	MH663997
fatp4	TGCCCCTCACATAGTTGCTG	CACTTCCTCGAACATCCCTCAT	MG637279
i-fabp	GACGGCACTGTGCTTAC	AAATCCTCTTAGCGTTG	MG637280

	TGG	ACACCT	
znt1	CACAAATGCGGATAGTGGGA	GGTCACTTGGAGCAACTGAAAC	KY652749
znt5	AAGAAAGGACAGAAGGGGACG	ACCAAAGCGGAGCAGTCAAA	KY652750
znt7	GAACTCCACCTGCTCTTGACC	CCGCCACATCTATCTGAACG	KY652751
zip4	CATTCATAACTTCGCAGACGG	CCAGAAAGCAACCCCAGATT	KY652752
mt	ATCCTTGCGAGTGCTCCA	GCAGGAATCGCCCTTACAC	EU124661
mtf1	CGAGTTGATGTTGCAGAGCC	GAGGTATGGAGGAAAGAAGGGA	KY652754
β-actin	GGACTCTGGTGATGGTGTGA	CTGTAGCCTCTCTCGGTCAG	EU161066
rpl7	GGCAAATGTACAGGAGCGAG	GCCTTGTTGAGCTTGACGAA	KP938522
hprt	ATGCTTCTGACCTGGAACGT	TTGCGGTTCAGTGCTTTGAT	KP938523
tuba	TCAAAGCTGGAGTTCTCGGT	AATGGCCTCGTTATCCACCA	KP938526
b2m	GCTGATCTGCCATGTGAGTG	TGTCTGACACTGCAGCTGTA	KP938520
ubce	TCAAGAAGAGCCAGTGGAGG	TAGGGGTAGTCGATGGGGAA	KP938524
gapdh	TTTCAGCGAGAGAGACCCAG	ATGACTCTCTTGGCACCTCC	KP938521
18srrna	AGCTCGTAGTTGGATCTCGG	CGGGTATTCAGGCGAGTTTG	KP938527
elfa	GTCTGGAGATGCTGCCATTG	AGCCTTCTTCTCAACGCTCT	KU886307

Supplemental Table 3. Effect of dietary different Zn sources on growth performance and morphometrical parameters of juvenile *P. fulvidraco*.

	$ZnSO_4$	Nano-Zn
IBM	4.08±0.02	4.09±0.02
FBM	22.59±0.49	25.14±0.27
WG	453.7±11.5 a	515.1±2.9 b
SGR	2.44±0.03 a	2.60±0.01 b
FI	20.11±0.05	20.24±0.38
FCR	1.09±0.03 b	0.96±0.02 a
VSI	6.13±0.28 a	7.02±0.28 b

ISI	1.30±0.03	1.37±0.05
CF	1.43±0.03 a	1.69±0.03 b
Survival	97.78±1.11	92.22±4.44

Values are means ± SEM (n=3 replicate tanks. For WG, SGR, FI and FCR, 26-30 fish each tank; for VSI, ISI and CF, six fish each tank). Values with different letters within the same row are significantly different at $P < 0.05$; CF, condition factor; FCR, feed conversion rate; IBW (g $fish^{-1}$), initial mean body weight; FBW (g $fish^{-1}$), final mean body weight; ISI, intestinal somatic index; SGR, specific growth rate; VSI, viscerosomatic index; WG, weight gain.

WG (%) = 100 × (final mean body weight − initial mean body weight) / initial mean body weight.

SGR (% d^{-1}) = 100 × (ln (final mean body eight) − ln (initial mean body weight)) / day.

VSI (%) = 100 × (viscera weight) / (body weight).

ISI (%)= 100 × (intestinal weight) / (body weight).

CF = 100 × (live weight, g) / (body length, cm) $^{3.}$

FI (g $fish^{-1}$): feed intake.

FCR = dry food fed (g) / wet weight gain (g).

Survival =100 × (final fish number) / (initial fish number).

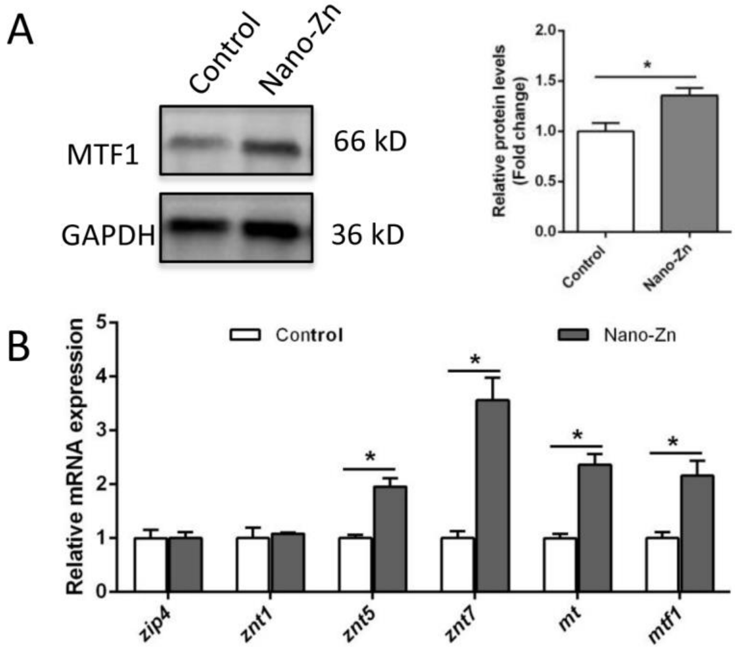

Supplementary Fig. 1. Intestinal epithelial cells didn't absorb Nano-Zn via caveolae-dependent and macropinocytosis pathways. (A) Protein levels of MTF1 of intestinal epithelial cells after 40 μM Nano-Zn incubation for 12 h. (B) The mRNA levels of Zn transport protein after 40 μM Nano-Zn incubation for 12 h. Values are means ± SEMs, n = 3-6. Asterisks (∗) indicate significant differences between control and Nano-Zn group ($p < 0.05$, n= 3).

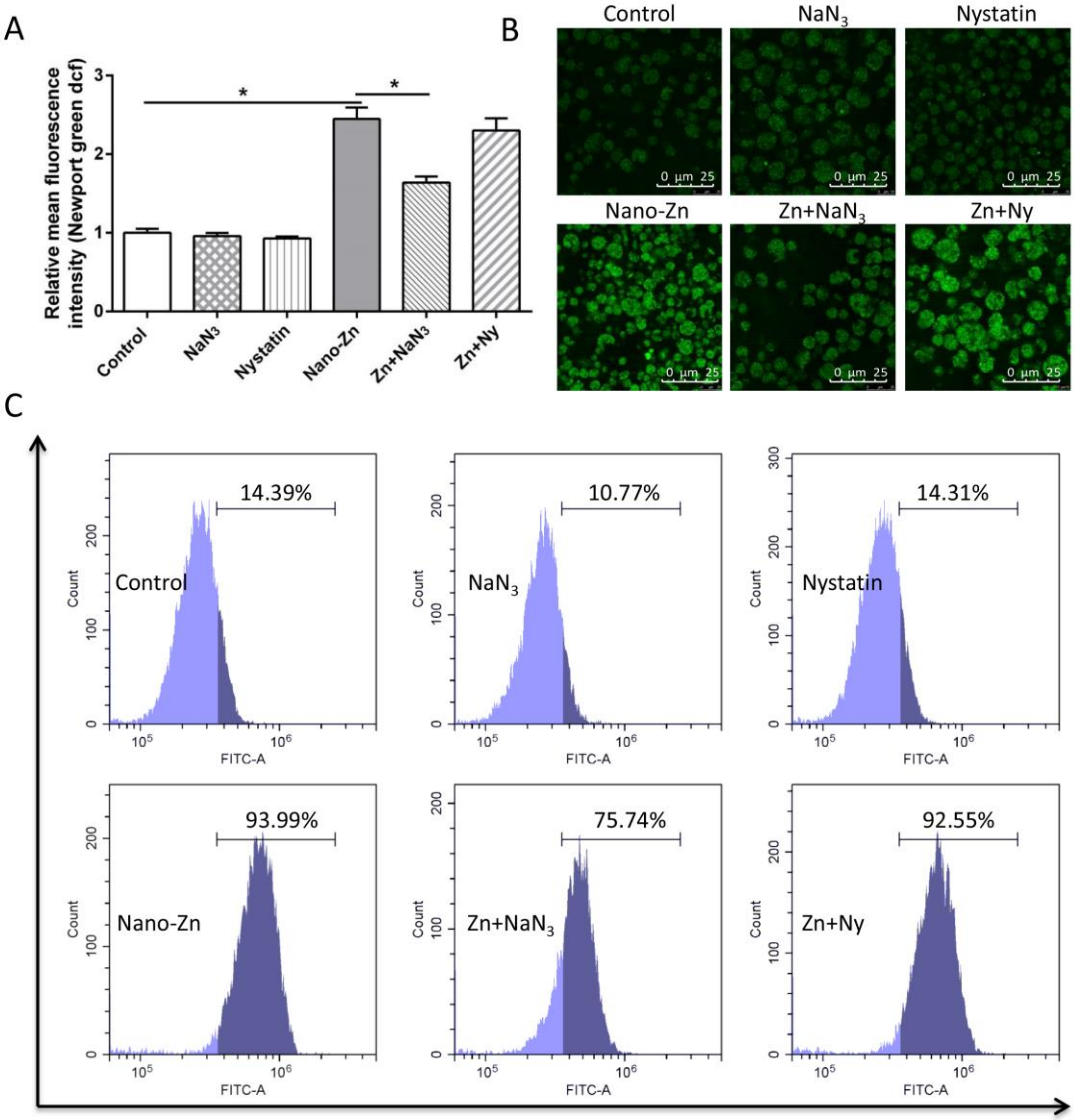

Supplementary Fig. 2. Nano-Zn absorption is energy-consuming in the intestinal epithelial cells. (A) Free Zn^{2+} was quantified by calculating FL1 (green) mean fluorescence intensity of intestinal epithelial cells incubated for 12 h in 40 μM Nano-Zn with 2-h 10 mM NaN_3 or 5μg/ml nystatin pretreatment. (B) Representative confocal microscopy stained with Zn^{2+} fluorescent probe (Newport green dcf). The primary intestinal epithelial cells from *P. fulvidraco* were incubated for 12 h in control or 40 μM Nano-Zn containing medium with or without 2 h 10 mM NaN_3 or 5μg/ml nystatin pretreatment. (C) The presence of DCF-stained Zn^{2+} was demonstrated by flow cytometric analysis of green (FL1) fluorescence intensity. The primary intestinal epithelial cells from *P. fulvidraco* were incubated for 12 h in control or 40 μM Nano-Zn containing medium with or without 2 h 10 mM NaN_3 or 5μg/ml nystatin pretreatment. Values are means ± SEMs, n = 3-6. Asterisks (∗) indicate significant differences between two groups ($p < 0.05$, n= 3). NaN_3, Sodium azide; Ny, Nystatin.
